# Loss of Dab2 Expression in Breast Cancer Cells Impairs Their Ability to Deplete TGF-β and Induce Tregs Development via TGF-β

**DOI:** 10.1371/journal.pone.0091709

**Published:** 2014-03-17

**Authors:** Shuguang Xu, Jingzhi Zhu, Zhiyong Wu

**Affiliations:** Department of Surgery, Renji Hospital, School of Medicine, Shanghai Jiaotong University, Shanghai, P. R. China; University of Alabama at Birmingham, United States of America

## Abstract

Dab2 is a multifunctional adapter protein which is frequently under-expressed in a variety of cancers. It is implicated in many critical functions, including several signaling pathways, cell arrangement, differentiation of stem cells, and receptor endocytosis. Transforming growth factor-β (TGF-β) is a secreted multifunctional protein that controls several developmental processes and pathogenesis of many diseases. It has been documented that Dab2 played an important role in TGF-β receptors endocytosis. Here, we present evidence that re-expression of Dab2 in SK-BR-3 cell partially restored its ability to deplete TGF-β in surrounding medium by normalizing the trafficking of TGF-β receptors. We also demonstrate that the difference in TGF-β depletions produced by Dab2 expression was sufficient to impact on the conversion of naive CD4+ T cells to regulatory T cells (Tregs), and thus inhibited the proliferation of T cells. This work revealed a critical result that breast cancer cell was deficient in Dab2 expression and related receptor endocytosis-mediated TGF-β depletion, which may contribute to the accumulation of TGF-β in tumor microenvironment and the induction of immune tolerance.

## Introduction

Breast cancer is a major risk to the health of women, accounting for 23% of annual new cancer cases, and 14% of deaths due to malignancies in women [1]. Despite various therapeutic options against this malignancy, more effective strategies for breast cancer treatment are needed, and their development may be aided by greater understanding of the molecular mechanisms underlying the pathogenesis of breast cancer.

Human Disabled-2 (*Dab2*) gene encodes a 96 kDa protein that itself has no catalytic activity but exerts its regulatory effects upon binding to other proteins [2]. Dab2 is involved in several signaling pathways, including TGF-β, Wnt, RAS/MAPK, and Src [3], [4], [5], and plays important roles in cell arrangement, cytoskeleton assembly, differentiation of stem cells [6], epithelial–mesenchymal transition [7]. *Dab2* is a tumor suppressor gene expressed in a variety of normal tissues. The expression of Dab2 has found to be decreased in several cancers [8], [9] including ovarian cancer, prostate cancer, etc. Conversely, ectopic expression of Dab2 inhibits the growth of prostate cancer, and choriocarcinoma cell lines [10], [11]. Despite of years of intensive studies, the role of Dab2 down-regulation in the development and progression of breast cancer is not fully defined.

The role of TGF-β in the occurrence and development of malignancies is microenvironment-dependent [12]. *In vitro,* studies also revealed that TGF-β can induce Foxp3 expression in CD4+CD25– naïve T cells, which then differentiate into regulatory T cells (Tregs) [13]. These Tregs may suppress effector T cell proliferation, leading to the formation of immune tolerance in the tumor microenvironment. The activation of the classic TGF-β signaling pathway is initiated by the binding of TGF-β to TβRII, followed by the activation of TβRI, Smad2/3 phosphorylation, formation of the Smad2/3 and Smad4 complexes, cultivating the entering of the Smad complex into the nucleus to regulate gene expression and ultimately cell growth and tumorogenesis [14], [15]. It has been clearly documented the interrelation between endocytic and signaling machineries in regulating TGF-β action [16], [17], [18]. In this process, receptor endocytosis can take place constitutively or be activated by ligand [19], [20]. TGF-β receptors are internalized into the early endosomal compartment of the cells, followed either by recycling back to the plasma membrane or by lysosomal degradation [18], [21], [22]. Clarke found that mink lung epithelial cell (MLEC) could deplete TGF-β by a TβRII-dependent mechanism involving receptor internalization [23]. Moreover, the time of complete depletion was consistent with that of Smad signaling. Thus, cells expressing TGF-β receptors may sense TGF-β in the condition medium, triggering TGF-β depletion by receptor trafficking. It has been hypothesized that deficient in receptor-dependent TGF-β depletion may contribute to the accumulation of TGF-β in the microenvironment.

The binding of Dab2 to TβRI and TβRII may aid the transmission of TGF-β signaling from the receptors to the Smad family of transcriptional activators [4]. Dab2 is a cargo specific adaptor protein that facilitates the assembly by coordinating cargo selection and lattice polymerization [24], [25]. Dab2 has also been shown to play an important role in the TGF-β receptor trafficking from early endosomes to recycling endosomes [26]. Under normal conditions, TGF-β receptors are transiently present in early endosomes. But, when Dab2 is deficient, receptors may stall in early endosomes and interrupt the transfer to recycling endosomes. These findings suggest that underexpression of Dab2 in cancer cells may result in abnormal TGF-β depletion. On the other hand, restoring normal Dab2 expression in Dab2-deficient cancer cells could normalize receptor recycling and TGF-β depletion. In conjunction with the overproduction of TGF-β in tumor cells [27], the loss of Dab2 expression and subsequent impairment of receptor-dependent TGF-β depletion may contribute to the accumulation of TGF-β in the microenvironment, a scenario that correlates with poor prognosis of cancer patients. To test this hypothesis in the context of breast cancer, here we investigated the effects of restoring Dab2 expression in SK-BR-3 cell (a human breast cancer cell line lacking Dab2 expression) on the TGF-β depletion, and the influence of abnormal TGF-β depletion on the differentiation of Tregs under *in vitro* conditions mimicking the tumor environment.

## Materials and Methods

### Patients and Ethics

Formalin-fixed paraffin-embedded breast tumors (including 72 invasive ductal carcinoma, 1 invasive lobular carcinoma, and 2 ductal carcinoma *in situ* with microinvasion) and normal breast tissues were selected from the archives at the Department of Surgery at Renji Hospital, Shanghai JiaoTong University School of Medicine (Shanghai, China), from 2010 to 2011. Peripheral blood mononuclear cells (PBMC) were obtained from healthy donors. The study was approved by the Independent Ethics Committee of Renji Hospital, Shanghai JiaoTong University School of Medicine, and written informed consent was obtained from all patients and healthy donors.

### Cell culture and condition medium collection

The breast cell lines (MDA-MB-231,SK-BR-3,MCF-7 and MCF-10A) were purchased from the Type Culture Collection of the Shanghai Institute for Biological Sciences, Chinese Academy of Sciences, Shanghai, China. The three breast cancer cells were grown in RPMI-1640 (GIBCO, Invitrogen, USA) supplemented with 10% FBS and penicillin/streptomycin (1∶100), in 5% CO_2_ at 37°C. The non-malignant mammary epithelial cell line MCF-10A was grown in DMEM supplemented with 10% FBS, 5 ug/ml crystallized bovine insulin, 10 ng/ml epidermal growth factor,100 ng/ml cholera toxin. The MLEC, stably transfected with an expression construct containing a truncated PAI-1 promoter fused to the firefly luciferase reporter gene,was a generous gift of Professor Rifkin (Department of Cell Biology, New York University Medical Center) [28]. MLEC cells were cultured in DMEM, containing 250 μg/μl of Geneticin (G-418 sulfate) in addition. Condition mediums were collected at different time points after cells exposed to the initial doses of TGF-β, and debris was removed by centrifugation at 1500 g for 10 min, and then stored at −80°C for later use.

### Plasmids, antibodies, and reagents

The Dab2 expression vector, pcDNA3.1(+)/Dab2 consisting of the full-length Dab2 gene inserted into the pcDNA3.1(+) expression vector (Invitrogen, Carlsbad, CA), was a gift from Joanna H. Tong (Department of Anatomical and Cellular Pathology, the Chinese University of Hong Kong, Hong Kong, PR China) [29]. Recombinant human TGF-β (isoform 1) was obtained from R&D Systems. Anti-phospho-Smad2 and anti-Smad2 antibody were used at a dilution of 1∶1000 dilution (Cell Signaling Technology, Danvers, MA). Anti-glyceraldehyde 3-phosphate dehydrogenase (GAPDH) antibody (Ab-mart, Shanghai, China) was used at a dilution of 1∶5000. Anti-Dab2 antibody (abcam ab76253, USA) was used at a dilution of 1∶2000, and the observed band size is 105 kDa according the product manual. Secondary antibodies against rabbit and mouse immunoglobulin G (Li-Cor, Lincoln, NE) were used at a dilution of 1∶10,000.

### Luciferase reporter gene activity assay

For the luciferase reporter gene activity assay, MLEC cells were seeded in 96-well plates (20,000 cells/well), lysed with 20 μl of passive lysis buffer (Promega, Madison, WI), frozen at −80°C for 2 h, and then incubated at 4°C for 10 min. The sample was stored at −20°C or brought to room temperature for assaying luciferase activity. Twenty μl of lysate was combined with 100 μl of substrate (Promega), and the resulting luminescence was read using a Biotek Synergy 4 luminometer. The linear dynamic range of the assay was confirmed in control experiments using serial dilutions of concentrated lysate.

### TGF-β reporter assay

A TGF-β reporter assay was used to measure TGF-β concentration in the condition medium as previously described [28]. The assay uses MLEC reporter cells, which express luciferase in a TGF-β dose-dependent manner to measure TGF-β concentration remaining in the condition medium from a separate group of test cells. At the end of an experimental treatment, a mixture of 50 μl of test cell medium with 50 μl of DMEM was transferred to 96-well plates seeded with the MLEC reporter cells (20,000 cells/well). In parallel, media containing known concentrations of TGF-β were added to a separate group of reporter cells in order to generate a standard curve. The MLEC cells were incubated for 24 h and processed for luciferase measurement.

### Restoration of Dab2 expression in SK-BR-3 cells

pcDNA3.1(+)/Dab2 expression vectors were transfected into SK-BR-3 cells using FuGENE HD (Roche, Mannheim, Germany). Restoration of Dab2 expression was confirmed by western blot analysis using Dab2 antibody (1∶4000, Santa Cruz Biotechnology, Santa Cruz, CA).

### Immunohistochemistry

Immunohistochemistry(IHC) was performed on the Ventana NexES automated stainer according to the manufacturer protocol. Anti-Dab2 primary antibodies from Abcam was used at 1∶50. The IHC was examined and imaged using an OLYMPUS BX51 microscope (Tokyo, Japan) at 1∶200. The scoring was independently assessed by two pathologists.

### Western blot analysis

Briefly, cells were lysed in RIPA buffer [50 mM Tris/HCl(pH 8.0), 1% Nonidet P40, 0.5% deoxycholate, 0.1% sodium dodecyl sulfate (SDS), 150 mM NaCl, and Complete™ Protease Inhibitor (Roche)]. Total cellular proteins were resolved by SDS-polyacrylamide gel electrophoresis (PAGE) and transferred to polyvinylidene difluoride (PVDF) membranes (Bio-Rad). IRDye 800CW secondary antibodies were used for visualization of specific protein bands.

### Transferring recycling

SK-BR-3 cells, grown on coverslips, were transfected with pcDNA3.1 (+)/Dab2 or empty vector using Fugene HD transfection reagent for 48 h, serum starved for 2 h in phenol-red-free DMEM, and then pulsed with Alexa Fluor 594-conjugated Tfn (25 μg/ml) for 5 min at 37°C. After multiple washing steps with ice-cold PBS to remove unbound Alexa 594–Tfn and inhibit transport, the coverslips were incubated with complete growth medium pre-warmed to 37°C for the time periods indicated, quickly washed with ice-cold PBS, fixed in 4% formaldehyde, mounted, and visualized using a LEICA TCS SP5 laser scanning confocal microscope.

### Real time PCR by SYBR green

PAI-1 mRNA expression was performed using the SYBR Premix Ex Taq™ (Takara) according to the manufacturer's instructions. Amplification reactions were performed by primers specific for PAI-1 (forward, 5′-TCTGCAGACCTGGTTCCCAC-3′; reverse, 5′-AGCCCCGTAGTTCCATCCTG-3′). The relative quantity of the PAI-1 mRNA was normalized to the level of the internal control GAPDH mRNA level. Triplicate measurements were made of all genes in each sample.

### Primary blood cells

Blood was obtained from healthy donors. Peripheral blood mononuclear cells were harvested by means of Ficoll–based density gradient(Axis-shield) centrifugation at 1,800 rpm for 20 min. Buffy coats were taken off and washed twice with PBS containing 2 mmol/L EDTA.

### Isolation of CD4+ Naïve T-cell subsets

For the isolation of naïve T cells, the magnetic cell separation technology MACS (Miltenyi Biotec) was used according to the manufacturer's instructions. CD4 + naïve T cells were negatively selected.

### Induction and neutralization experiments

The purified naïve CD4+ T cells (2×10^5^) were cultured with 200 ul conditioned medium described above in 96-well plates at 37°C and 5% CO_2_ plus CD28 antibody (10 μg/mL; eBioscience) and IL-2 (50 IU/mL; eBioscience). For activation of lymphocytes, the plates were coated with PBS containing 5 μg/mL CD3 antibody (eBioscience) at 4°C overnight. After 72 h of cultivation, the proportion of CD4 + Foxp3 + T cells was detected by FCM. A panspecific TGF-β antibody (R&D) (3 ug/ml) was used for neutralization of TGF-β in the condition medium.

### CFSE-based suppressor assay

The carboxyfluorescein diacetate succinimidyl ester (CFSE) dilution assay was performed for the proliferation inhibition assay. Briefly, the CD4 + T cells from induction by TGF-β were pooled with CFSE-labeled CD4 + naïve T cells in 1∶1 ratio (1×10^5^ each) and seeded in 96-well plates in the presence of 10 μg/mL CD28 antibody. The plates were coated with PBS containing 5 μg/mL CD3 antibody described above. After 4 d, the cells were harvested and proliferation was measured by loss of CFSE dye with flow cytometry.

### Statistical analysis

Descriptive numerical data were given as mean ± SD. Analysis of variance (ANOVA) was applied to analyze between-group differences with statistical adjustments; categorical data were analyzed using the x^2^ test. All statistical procedures were performed with the SPSS 16. Values of P less than 0.05 were considered statistically significant.

## Results

### Deficient expression of Dab2 in breast cancer and breast cancer cell lines

**Figure 1:**
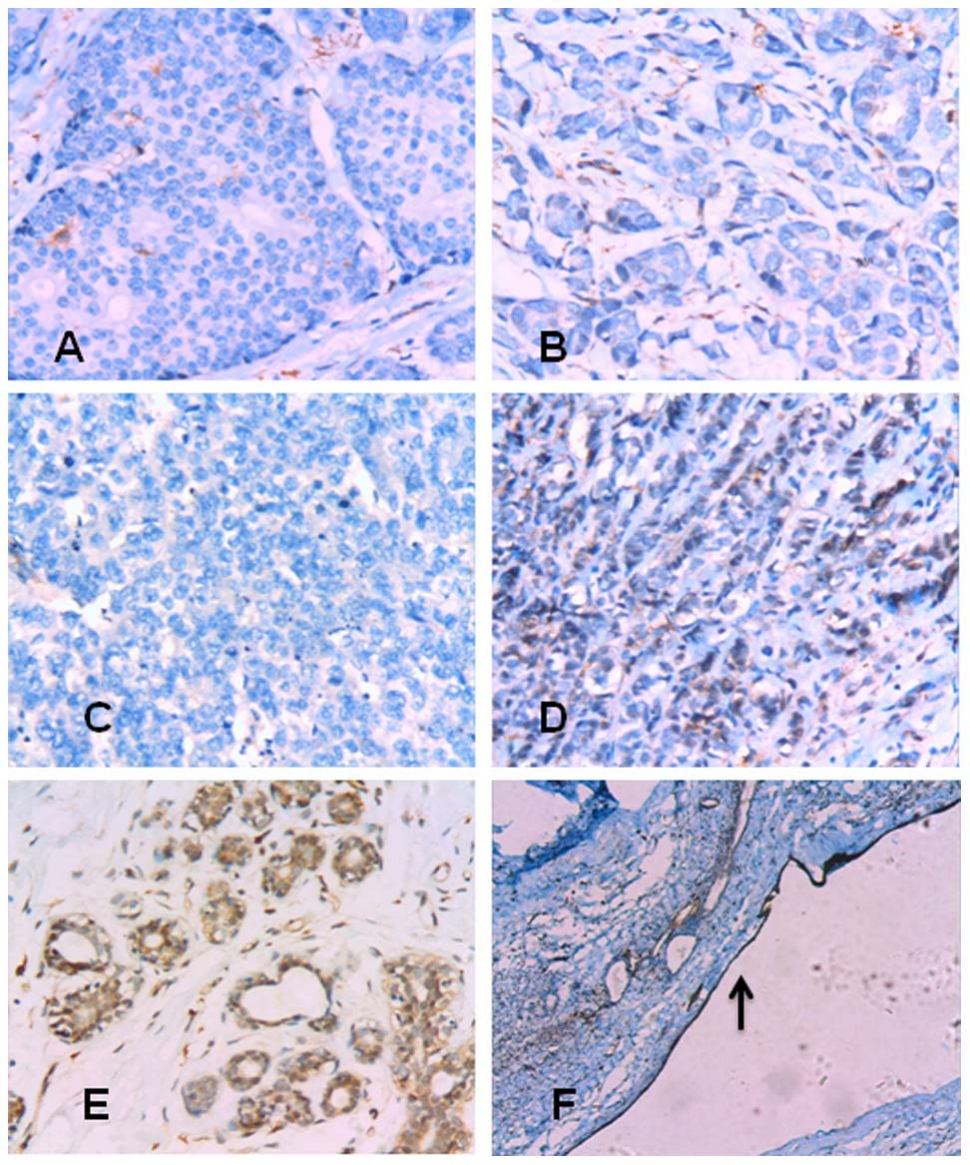
Expression of Dab2 in breast cancer specimens. (A) Ductal carcinoma *in situ* with microinvasion showing underexpression of Dab2 (original magnification ×200). (B) Invasive ductal carcinoma showing underexpression of Dab2 (×200). (C) Invasive lobular carcinoma showing underexpression of Dab2 (×200). (D) Medullary carcinoma cells showing partial Dab2 positivity (cytoplasm; 2+) (×200). (E) Normal breast epithelial cells showing strong Dab2 positivity in the cytoplasm (×200). (F) Epithelial cells in a galactocele served as positive control (arrows indicate positive cells) (×50).

Breast cancer tissue specimens (invasive ductal carcinoma [n = 72]; invasive lobular carcinoma [n = 1]; medullary carcinoma [n = 1]; ductal carcinoma *in situ* with microinvasion [n = 2]) were collected from 76 patients and subjected to immunohistochemistry for Dab2, together with normal breast tissues from 10 controlled subjects. The expression of Dab2 was totally lost in 76% (58/76) of specimens, and focally positive but reduced in the remaining 18 (24%) specimens. Representative photomicrographs were shown in Figure 1A-D. Normal breast tissue specimens from 10 subjects were stained strongly positive for Dab2 as shown in Figure 1E. Of note, the expression of Dab2 was also diminished in ductal carcinoma *in situ* with microinvasion.

**Figure 2:**
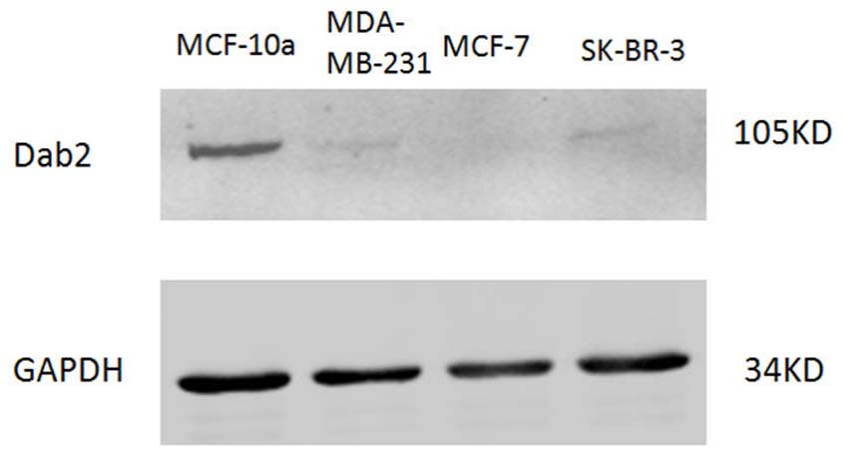
Dab2 protein expression in three breast cancer cell lines demonstrated by western blots. Western blot analysis of Dab2 protein expression in breast cancer cell lines. None of the 3 breast cancer cell lines showed detectable levels of Dab2 protein, while the nonmalignant mammary epithelial cell line MCF10A (positive control) showed the presence of Dab2 protein. Results were representative of 3 independent experiments.

The expression of Dab2 in three breast cancer cell lines (MCF-7, MDA-MB-231, and SK-BR-3) was analyzed with the western blotting assay. As shown in Figure 2, levels of Dab2 protein expression in all three breast cancer cell lines were reduced significantly, compared to normal breast epithelial cells (MCF-10A; the positive control).

### TGF-β depletion in SK-BR-3 cells

**Figure 3:**
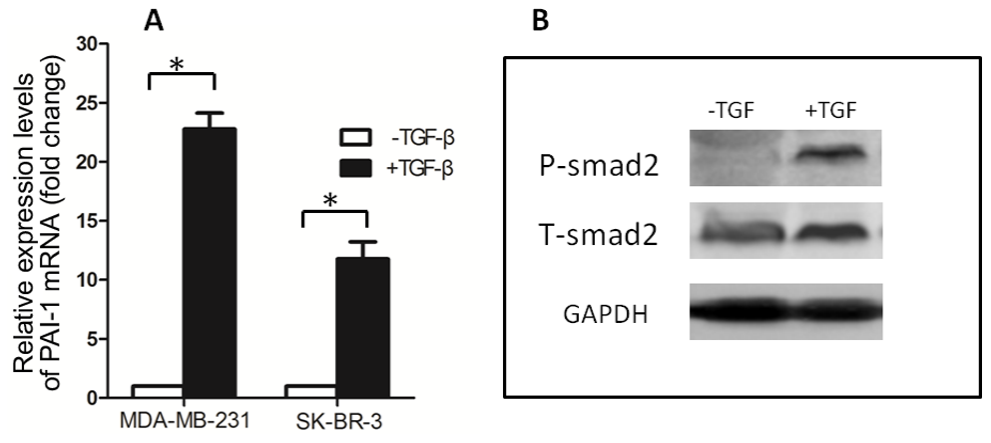
The sensitivity of breast cancer cell lines to TGF-β. (A) Induction of PAI-1 mRNA by TGF-β in breast cancer cell lines. MDA-MB-231 and SK-BR-3 cells were treated for 2 h with or without TGF-β (100 pM) before isolation of total RNA with Trizol. PAI-1 mRNA levels were determined with RT-Quantitative PCR. Data are means±SD of at least three independent experiments in each condition. * P<0.05. (B) TGF-β induced phosphorylation of Smad2 in SK-BR-3 cells. SK-BR-3 cells in complete medium was treated for 30 min with or without TGF-β(100pM) before being lysed with SDS sample buffer. The level of Phospho-Smad2 was analyzed by immunoblotting.

The ability of SK-BR-3 cells to deplete TGF-β was quantified by measuring the TGF-β concentration in condition medium with a TGF-β reporter assay. Therefore we first tried to verify the responsiveness of breast cancer cells to exogenous TGF-β by detecting the transcription of PAI-1, a target gene of TGF-β [30], after TGF-β treatment (100 pM for 2 h). As shown in Figure 3A, upon treatment with TGF-β, the SK-BR-3 and MDA-MB-231 showed variable increases in expression of PAI-1 mRNA. Furthermore, identical treatment with TGF-β (100 pM for 30 min) significantly increased the phosphorylation of Smad2 in the SK-BR-3 cells (Figure 3B). These results suggested that these breast cancer cells maintained functional TGF-β receptors and the Smad2 signal transduction pathway.

**Figure 4:**
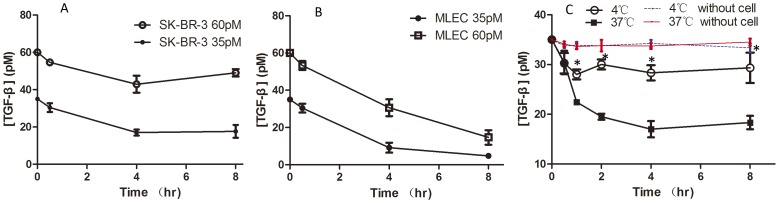
Time course curves of TGF-β depletion by SK-BR-3 and MLEC cells under different incubation conditions. SK-BR-3 and MLEC cells were incubated in condition medium containing 35 or 60 pM of TGF-β for up to 8 h and aliquots of the incubation medium were collected at different time points. The remaining TGF-β content in those samples was quantified by a TGF-β reporter assay. (A) Curves of TGF-β depletion in SK-BR-3 cells in the presence of TGF-β at 35 and 60 pM. (B) Curves of TGF-β depletion in MLEC cells in the presence of TGF-β at 35 and 60 pM. (C) Curves of TGF-β depletion in SK-BR-3 cells at 4°C and 37°C (35 pM TGF-β). At 4°C, endocytosis was stopped. Data are Means±SD of at least three independent experiments in each condition. * P<0.05.(4°C vs 37°C)

To evaluate the ability to deplete TGF-β, SK-BR-3 cells (seeded in 6-well plates, 8×10^5^/well, maintained in 2 ml of medium) was exposed to the initial doses of 35 and 60 pM respectively for up to 8 h. Condition mediums were collected at different time points and the concentrations of remaining active TGF-β were measured by MLEC cells with stable expression of Luciferase reporter gene. The TGF-β content in the SK-BR-3 cell condition medium declined from its initial concentrations continuously during the first 4 h of incubation and then stabilized for the remaining 4 h. Even after 8 h of incubation, SK-BR-3 cell was unable to completely deplete TGF-β presented in the surrounding media (Figure 4A). Under the same conditions, TGF-β was almost completely depleted by MLEC, which is sensitive to TGF-β, after 8 h of TGF-β treatment (Figure 4B). Although the ability to remove TGF-β could not be compared between the two cell types, it was clear that SK-BR-3 cells were less capable of removing TGF-β from the medium.

Cells can deplete medium TGF-β by (i) binding TGF-β to TβRII and then undergoing clathrin-dependent endocytosis, and (ii) reversible binding to the cell surface [23]. To elucidate the relative contribution of these two mechanisms for TGF-β depletion, SK-BR-3 cells were incubated at 4°C to block the endocytosis without inhibiting TGF-β binding to the cell surface. As expected, a partial depletion of TGF-β occurred at 4°C during the first 30 min. Whereas at 37°C the removal of TGF-β lasted for 4 h and to a much greater degree (Figure 4C), hence endocytosis played a more important role in TGF-β depletion in SK-BR-3 cells than the reversible binding of TGF-β to the cell surface. As the incubation of SK-BR-3 cells at 37°C continued beyond 4 h, there was no further decline in medium TGF-β content. We then suspect that SK-BR-3 cells not only have attenuated TGF-β depletion, but also up-regulation of TGF-β expression, like other cancer cells [27].

### Influence of Dab2 on TGF-β depletion in SK-BR-3 cells

**Figure 5:**
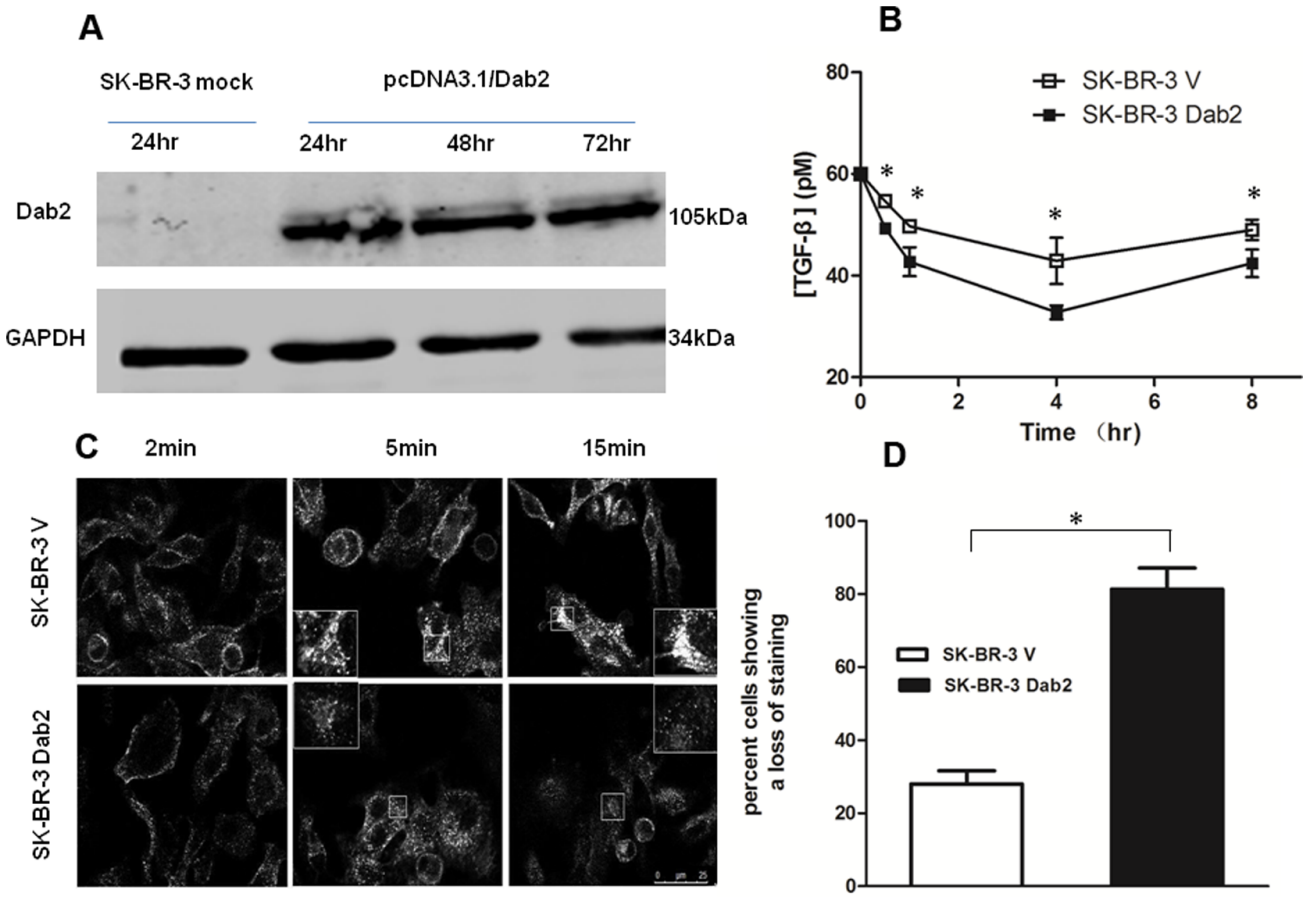
Influence of Dab2 expression on TGF-β depletion in SK-BR-3 cells. (A) Western blot analysis depicting Dab2 expression in SK-BR-3 cells after transfection with pcDNA3.1/Dab2. (B) TGF-β depletion by mock- and Dab2-transfected SK-BR-3 cells were measured with the reporter assay. Re-installation of Dab2 partially restored TGF-β depletion in the SK-BR-3 Dab2 cells, indicated by significant lower TGF-β content in its condition medium compared with the condition medium of SK-BR-3 V cells. (C) Influence of Dab2 on recycling of Tfn in breast cancer SK-BR-3 cells. Bar, 25 um. (D) After endocytosis, Tfn accumulates in the cytoplasm around the nucleus. The proportion of cells that showed diffued staining(indicative of Tfn recycling) within 15 min, out of a total of 50 cells examined, was calculated. *P<0.05.

We presumed the loss of Dab2 expression could contribute to the incomplete removal of TGF-β by the SK-BR-3 cell. To directly evaluate the effect of Dab2 on endocytosis-mediated TGF-β depletion, we tried to restore the expression of Dab2 in the SK-BR-3 cells by transient transfection with the pcDNA3.1(+)/Dab2 vector (named the new cell as SK-BR-3 Dab2). The re-expression of Dab2, as revealed by the western blot analysis, was detected at 24 h after transfection and reached a stable level at 72 h (Figure 5A). We measured the time courses of TGF-β depletion in the condition medium of mock-transfected cells (named the new cell as SK-BR-3 V) and SK-BR-3 Dab2 cells. When exposed to an initial dose of 60pM TGF-β, the TGF-β level remained in the condition medium of SK-BR-3 Dab2 cells after 4 h of incubation was 25% lower (12pM) than that of SK-BR-3 V cells. For uncertain reasons, the levels of TGF-β in the condition medium of both mock- and Dab2 transfected cells increased slightly at the 8 h time point (Figure 5B). Therefore, although re-expression of Dab2 significantly augmented TGF-β depletion, TGF-β was not completely depleted from the medium under this experimental condition, indicating that other reasons may also contribute to the attenuated TGF-β depletion in this breast cell line.

Transferrin (Tfn) is a marker commonly used in the detection of endocytosis. After endocytosis, Tfn is recycled back to the plasma membrane in a Rab4-, Rab35-, or Rab11-dependent manner [31], [32]. Tfn remains associated with the receptor until it is recycled to the cell surface and is released as apotransferrin, and therefore the loss of cell-associated fluorescence over time indicates recycling efficiency. Rab11-dependent pathway is also required for the recycling of TGF-β receptors [18], [26]. Thus, Tfn endocytosis was employed to detect changes in the Rab11-mediated pathway in SK-BR-3 cells after they re-gained Dab2 expression. As shown in Figure 5C, the internalized Tfn was accumulated in enlarged or swollen perinuclear endocytic compartment of the Dab2-deficient SK-BR-3 V cells when stained for 15 minutes. In contrast, much smaller and weaker Tfn stained granules were observed in the Dab2-expressing SK-BR-3 Dab2 cells. The proportion of cells with diffused Tfn staining was increased by 55% in SK-BR-3 Dab2 compared with SK-BR-3 V cells (Figure 5D). The results suggested that the Tfn recycling was markedly improved in SK-BR-3 Dab2 cells after re-expressed Dab2.

### TGF-β depletion of SK-BR-3 cells affect differentiation of Tregs, which exert suppressive effects on T cells

**Figure 6:**
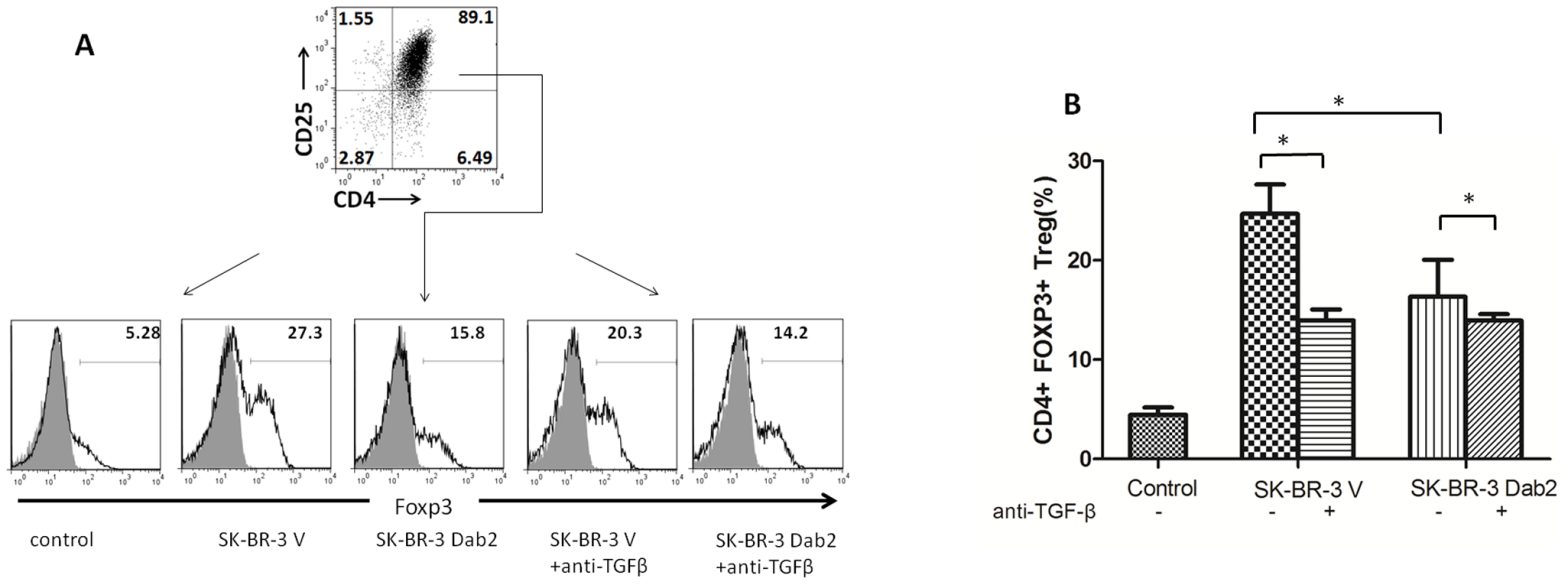
Differentiation of CD4+CD25- naïve T cells from peripheral blood of healthy subjects. (A) CD4+CD25- naïve T cells treated with negative control medium, condition medium from SK-BR-3 V cells (4 h), and condition medium from SK-BR-Dab2 cells (4 h). Cells were treated in the presence or absence of TGF-β antibody, harvested 96 h later, and analyzed by flow cytometry for CD4+CD25+Foxp3+ Tregs. Representative data from one determination are shown. (B) Proportion of CD4+CD25+Foxp3+ Tregs formed in different groups. Data are means ± SD of three independent experiment (*P<0.05).

Studies with the SK-BR-3 Dab2 cells described above clearly indicate that the re-expression of Dab2 restore the cells ability to deplete TGF-β in surrounding medium via accelerated endocytosis. After treatment with 60 pM TGF-β for 4 h, the TGF-β content in the condition medium of SK-BR-3 Dab2 cells was 25% lower than that of SK-BR-3 V cells, which translates to approximately 12 pM (300 pg/ml). Next, we investigated whether the alteration in TGF-β depletion affects the differentiation of CD4+ T cells into Tregs and the subsequent activities and functions of immune cells. To that end, the condition medium collected from cell cultures of SK-BR-3 Dab2 cells or SK-BR-3 V cells exposed to TGF-β for 4 h were used to treat naïve T (CD4+ CD25–) cells from peripheral blood of healthy subjects. The proportion of Tregs (CD4+ CD25+ Foxp3+) was detected by flow cytometry. As expected, the SK-BR-3 V condition medium (with more residual TGF-β) induced a greater number of Tregs than the condition medium of the SK-BR-3 Dab2 (Figure 6). Further supporting the action of TGF-β in the condition medium in the formation of Tregs, the addition of a neutralizing monoclonal antibody against TGF-β significantly compromise the differentiation of Tregs (Figure 6). Our data indicated that TGF-β in the condition medium played a certain role in the conversion of naïve T cells into Tregs.

**Figure 7:**
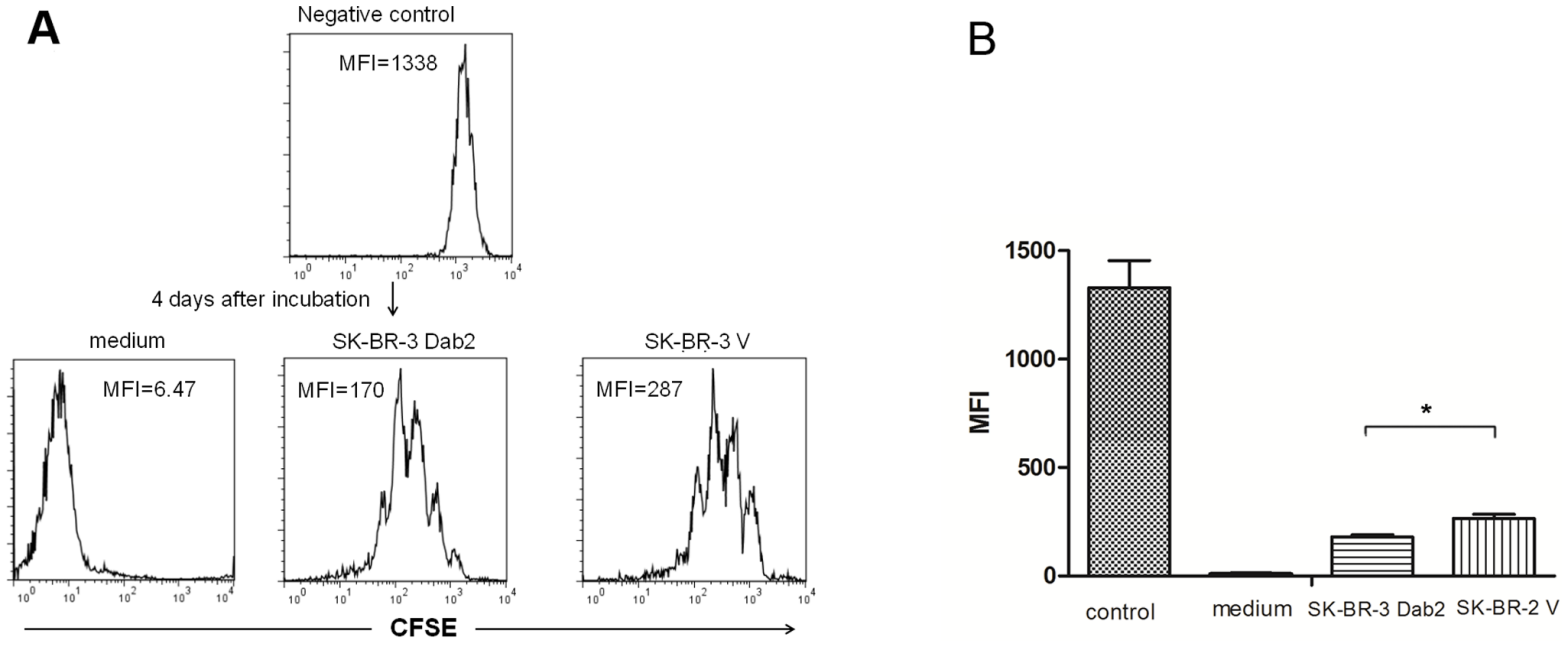
The inhibitory effects of Tregs induced with condition mediums from mock- or Dab2-transfected SK-BR cells on T cell proliferation. Condition medium from SK-BR-3 V cells or SK-BR-Dab2 cells were used to differentiate CD4+ CD25-naïve T cells to CD4+CD25+Foxp3+Tregs. The freshly differentiated Tregs were then co-cultured with CFSE-labeled CD4+CD25- naïve T cells at a ratio of 1:1 to examine the effects of Tregs on T cell proliferation. A lower mean fluorescence intensity (MFI) indicates more cells proliferation in this assay (CFSE label will be diluted 50% each time cell divides). (A) Representative data from 1 of 3 assays (medium-treated group, SK-BR-3 V medium group, and SK-BR-3 Dab2 medium group were treated with anti-CD3/CD28; negative control cells were untreated). (B) Means±SD of three independent experiments (MFI) in different groups (*P<0.05).

It has been reported that Foxp3 can be transiently up-regulated in activated CD4+ lymphocytes without leading to differentiation into functional Tregs [33]. We therefore assessed the function of TGF-β-induced Tregs in a suppressor assay. To this end, freshly induced Tregs were co-cultured in a 1∶1 ratio with freshly isolated naïve CD4+ T cells, and treated with CD3- and CD28-specific antibodies to induce T-cell activation. Cell proliferation was measured at day 4 by CFSE (Figure 7). A loss of mean fluorescence intensity (MFI) of CFSE indicates cell proliferation in this assay. Compared with control medium, the MFI was markedly higher in Treg: naïve CD4+ T-cell cocluture system, suggesting the two groups of Tregs had a suppressive effect on T-cell activation. In addition, the MFI of co-culture with Tregs induced by SK-BR-3 V cells condition medium was higher than that by SK-BR-3 Dab2 cells. This indicated that Tregs induced by condition medium of SK-BR-3 V cells (with more TGF-β) were more potent to inhibit the proliferation of naïve CD4+ T cells than the Tregs induced by the SK-BR-3 Dab2 cell medium (with less TGF-β).

## Discussion

### 1. Dab2 promotes receptor-dependent TGF-βdepletion through endocytosis of TGF-β receptor

Aberrant TGF-β signaling has been found to be associated with a variety of human diseases including malignancies. Breast cancer tissues express higher levels of TGF-β than normal breast tissues [34], [35], [36]. Furthermore, a significantly greater fraction of invasive carcinomas express immunodetectable TGF-β than the non-invasive ones [37], [38]. Plasma TGF-β level in breast cancer patients is closely related to the cancer burden [39], [40], [41]. However, the relationship between TGF-β expression and prognosis of breast cancer patients remains controversial. Some investigators found that high TGF-β expression predicts good prognosis [42], [43], [44], while others showed the opposite [38], [45], [46]. These evidences have encouraged us to investigate the mechanisms underlying TGF-β accumulation. Except for the upregulation TGF-β expression and secretion in cancer cells, what factors else contribute to the elevated TGF-β level is still unclear. By investigating the relationship between TGF-β depletion and phospho-Smad2 dynamics quantitatively, Clarke et al found that compromised TGF-β depletion may contribute to TGF-β accumulation in the microenvironment of cancers [23]. We postulate that increased TGF-β in cancers and peripheral blood is attributable not only to upregulation of TGF-β secretion by cancer cells and mesenchymal cells but also to compromised TGF-β depletion.

TGF-β depletion is dependent on receptor endocytosis. Originally discovered as a relatively simple process to transport molecules across the plasma membrane, endocytosis actually provides necessary spatial and temporal dimensions for the integration of signals, and thereby serves as a master organizer of cell signaling for almost all aspects of cellular function [22], [47]. Receptor-dependent endocytosis may influence the role of TGF-β in cancer cells [48]. Zi discovered that cells responded differently to continuous versus pulsating TGF-β stimulation [49], and ligand depletion was an important mechanism for terminating transient signaling and generating a long-term switch-like response. Endocytosis thus critically regulates cellular biological functions and the fate of cells [50].

**Figure 8:**
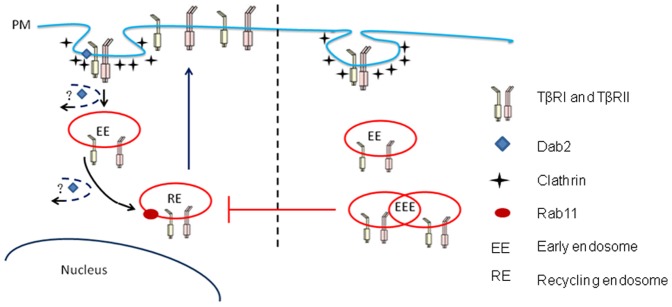
Proposed itinerary of TGF-β receptors in Dab2 absent cells or Dab2 re-expression cells. TGF-β receptors undergoes endocytosis via clathrin-coated pits with involvement Dab2. Receptors enter the early endosomes and return to the cell membrane via Rab11-positive recycling endosomes. Dab2 can promote the transfer of receptors from early endosomes to recycling endosomes. When Dab2 expression is deficient, the TGF-β receptor may accumulate in the early endosomes after endocytosis, leading to the interruption of receptor movement to recycling endosomes, which hinders the return of receptors to cell membranes.

**Figure 9:**
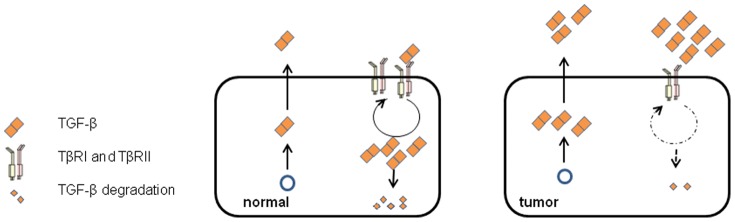
Model of how normal and cancer cells regulate the extracellular levels of TGF-β. The concentration of extracellular TGF-β is maintained by the balance between cellular production of TGF-β and depletion by the endocytosis. The underexpression of Dab2 and impaired TGF-β depletion in cancer cells lead to increased extracellular TGF-β concentration and cancer progression.

A study in 1980s using ^125^I-labelled TGF-β suggested that TGF-β was rapidly internalized via its receptors and degraded in lysosomes [51]. The optimal internalization of TGF-β requires both TβRI and TβRII [52]. Receptor endocytosis can take place constitutively, or be triggered by ligand binding [19], [20]. After entering the early endosomes, the receptors without ligand-bound are sorted to recycling endosomes and returned back to the plasma membrane. The ligand-bound receptors can continue their signaling activity in early endosomes. Some of them are recycled back to the plasma membrane for re-use, or sorted to late endosomes, where they are targeted to lysosomes for degradation [18], [22]. Dab2 has been shown to associate with the TβRI and TβRII and modulate Smad activation [4]. Moreover, Dab2 plays an important role in TGF-β receptors endocytosis [4], [25], [53], [54]. For latter role, Dab2 facilitates the transfer of TGF-β receptor from early endosomes to recycling endosomes [26]. Loss of Dab2 leads to the accumulation of receptors (as well as Tfn) in aberrantly enlarged early endosomal antigen 1-positive endosomes, and a reduction of TGF-β receptors in the recycling endosomes by about 70%, significant disruption of receptors recycling to membrane. Because plasma membrane composition is determined by the balance between endocytic uptake and recycling of macromolecules [31], [32], we investigated whether Dab2 functioned in the TGF-β depletion process (Figure 8). In the present study, after introduction of Dab2 into SK-BR-3 cells, TGF-β receptor recycling was improved significantly and TGF-β depletion was recovered partly. Together with the up-regulation of TGF-β expression and secretion [27], a compromised depletion of TGF-β in surrounding medium due to the loss of Dab2 contribute to the accumulation of TGF-β in the tumor microenvironment. This condition facilitates cancer cells proliferation, invasion, and metastasis, leading to a poor prognosis (Figure 9).

Endocytosis of TGF-β receptors and its role in TGF-β signaling have been studied by multiple methods. To date, owing to the lack of good antibody for TGF-β receptors, tracing the trafficking of endogenous TGF-β receptors remains difficult. Investigators have constructed chimeric receptors consisting of the extracellular domain of the granulocyte/macrophage colony-stimulating factor (GM-CSF) alpha or beta receptor fused to the transmembrane and cytoplasmic domain of the type I or type II TGF-beta receptor. GM-CSF can specifically bind to the extracellular segment, and thus fluorescence labeled secondary antibody can be used to trace the receptor trafficking [55]. The present study focused on the influence of receptor recycling on the extracellular TGF-β concentration. Thus, the experimental design was simplified and the chimeric receptor system was not applied. In addition, the Rab11-related pathway is involved in the recycling of Tfn receptor and TGF-β receptor, and thus Tfn was used to examine changes in the Rab11-dependent pathway, which may also to some extent account for the changes in the TGF-β receptor recycling.

### 2. Dab2 can regulates Treg development via promoting TGF-βdepletion

The tumor microenvironment is crucial for tumor progression [56], [57]. The immune cells in the microenvironment lose their ability to kill cancer cells, and on the contrary, facilitate cancer cell growth, invasion, and metastasis [58]. Since the initial identification by Sakaguchi in 1995 [59], Tregs have been demonstrated to play pivotal roles in autoimmune tolerance and tumor escape from immunological control by suppressing the activation and proliferation of T cells, B cells, and natural killer cells [60]. The number of Tregs in patients with cancers inversely correlates with their survival rate [61], [62]. The increased number of Tregs in the peripheral blood of patients with gastrointestinal malignancies and esophageal cancer is associated with immunosuppression [63], [64]. The number of Tregs in the peripheral blood and cancer microenvironment increases markedly in patients with breast cancer [65]. TGF-β stimulates the formation of Tregs through the induction of Foxp3 expression [66]. Blockade of TGF-β­induced signaling with antibodies or genetic manipulation leads to decreased numbers of Tregs in some tumour­bearing animals [67], [68]. Therefore, reducing TGF-β in the tumor microenvironment could attenuate the immunosuppressive effects of Tregs, resulting in increased antitumor immunity. In the present study, co-culture system was employed to mimic the tumor microenvironment. After SK-BR-3 cells re-expressed Dab2, TGF-β depletion was improved, as demonstrated by the reduction of extracellular TGF-β concentration by about 12 pM after TGF-β treatment for 4 h. This degree of reduction in TGF-β concentration was found to reduce the differentiation of naïve CD4+ T cells into Tregs, and the suppression of T-cell proliferation by differentiated Tregs. As far as we know, the present study is the first to investigate the influence of Dab2 on TGF-β depletion and immune cell function.

Taken together, our findings indicated that Dab2 was largely lost in breast cancer cells. The loss of Dab2 expression may impair TGF-β depletion mediated by receptor endocytosis. The accumulation of TGF-β in the tumor microenvironment may promote the naïve CD4+ T cells to Tregs differentiation and development of immune tolerance. These findings suggested that targeting TGF-β depletion in the tumor microenvironment may represent a new strategy for the treatment of breast cancer.
